# Species richness and beta diversity patterns of multiple taxa along an elevational gradient in pastured grasslands in the European Alps

**DOI:** 10.1038/s41598-020-69569-9

**Published:** 2020-07-27

**Authors:** Veronika Fontana, Elia Guariento, Andreas Hilpold, Georg Niedrist, Michael Steinwandter, Daniel Spitale, Juri Nascimbene, Ulrike Tappeiner, Julia Seeber

**Affiliations:** 1 Institute for Alpine Environment, Eurac Research, Drususallee 1, 39100 Bozen, Italy; 2 Department of Ecology, University of Innsbruck, Sternwartestraße 15/Technikerstraße 25, 6020 Innsbruck, Austria; 3 Natural Sciences Museum of South Tyrol, via Bottai 1, 39100 Bolzano, Italy; 4 Department of Biological, Geological and Environmental Sciences, University of Bologna, via Irnerio 42, 40126 Bologna, Italy

**Keywords:** Ecology, Biodiversity

## Abstract

To understand how diversity is distributed in space is a fundamental aim for optimizing future species and community conservation. We examined in parallel species richness and beta diversity components of nine taxonomic groups along a finite space, represented by pastured grasslands along an elevational gradient. Beta diversity, which is assumed to bridge local alpha diversity to regional gamma diversity was partitioned into the two components turnover and nestedness and analyzed at two levels: from the lowest elevation to all other elevations, and between neighboring elevations. Species richness of vascular plants, butterflies, beetles, spiders and earthworms showed a hump-shaped relationship with increasing elevation, while it decreased linearly for grasshoppers and ants, but increased for lichens and bryophytes. For most of the groups, turnover increased with increasing elevational distance along the gradient while nestedness decreased. With regard to step-wise beta diversity, rates of turnover or nestedness did not change notably between neighboring steps for the majority of groups. Our results support the assumption that species communities occupying the same habitat significantly change along elevation, however transition seems to happen continuously and is not detectable between neighboring steps. Our findings, rather than delineating levels of major diversity losses, indicate that conservation actions targeting at a preventive protection for species and their environment in mountainous regions require the consideration of entire spatial settings.

## Introduction

Detecting and understanding dynamics and causes of biodiversity losses or gains have become a worldwide challenge in the field of nature conservation. However, in many regions on Earth not even the status quo of living organisms is known, often hindering reliable evidence on imminent risks and threats^1^. Aside financial and political reasons, comprehensive biodiversity assessments are often hampered by more trivial reasons: many taxa are almost unknown to science, others are difficult to detect, requiring sophisticated methods and equipment as well as high taxonomic expertise^2^. Thus, most studies focus on one or few taxonomic groups, inhibiting a holistic view on a coexisting community which usually strongly interacts within a given space^3^. Multi-taxon approaches looking at the main players in an ecosystem are rarely found but more and more required when trying to understand spatial dynamics of diversity at a superordinate level^4^. Encouragingly, the number of recently established (long-term) monitoring programs and networks is increasing steadily, promising a proliferation of knowledge on presence, co-occurrence and abundance of living animals and plants on a species level over broad spatial and temporal scales^5,6^.

Another difficulty in diversity research concerns the definitions of concepts and controversially discussed diversity measures, especially when referring to beta diversity^7^. Beta diversity is the linkage between local alpha diversity and regional gamma diversity and the first approach, proposed by Whittaker^8,9^ stated that beta results from the ratio of gamma to alpha. However, over the years, a series of more specific definitions and formulas have been developed [cf.^10,11^] and since the partitioning diversity forum in 2010^12^, the need for a measure of beta, independent of alpha and gamma, has been raised. Thereupon, research advanced rapidly and went deeper into partitioning the components of beta diversity^13–16^, but again differently developed approaches are hampering a wider understanding and comparison of findings. For example, Legendre and Caceres^16^ decompose diversity into species and site contributions representing a non-directional approach which determines variation in community structure among a set of sample units. In contrast, Baselga and Orme^17^, Podani and Schmera^15^, and Carvalho et al.^14^ stick to a directional approach along a predefined gradient performing pair-wise comparisons. Of the latter, Baselga and Orme^17^, who distinguish between turnover (i.e. species replacement between a pair of samples) and nestedness (i.e. species gain or loss between a pair of samples) components, developed the currently most widely used approach^18^. Generally, turnover is supposed to be the result of environmental, abiotic impacts or dispersal processes, while nestedness may reflect colonization and extinction patterns^18–20^.

Both components have been recognized to adequately detect spatial diversity patterns^18^ due to the underlying dissimilarity measures which compare species compositions between two sample units.

An ideal predefined space to study mechanisms which determine spatial variation of species assemblage is represented by elevational gradients along mountain slopes^21^. In advantage to latitudinal gradients, they encompass short geographical distances, attenuate biotic responses caused by historical or biogeographical reasons^22^ and facilitate the delimitation of changes in community structure due to environmental drivers^23^.

The main aim of our study is to uncover the spatial scaling of species communities of nine taxa along an elevational gradient and to detect parallelisms or dissimilarities between the taxonomic groups. We recorded presence and, where possible, abundance, of nine organism groups including lichen, bryophytes, vascular plants, ants, beetles, spiders, butterflies, grasshoppers, and earthworms occurring in alpine pastured grassland along a complete elevational gradient spanning 1,500 m. First, we compared species richness for all groups along the gradient and then applied the most widely used approach to partition beta diversity^18^, developed by Baselga in 2010^13^. We considered the two components turnover and nestedness at two levels: from the lowest elevation to all other elevations (along elevation), and between neighboring elevations (step-wise).

On the basis of previous literature [e.g.^24–26^], we expect that species richness decreases with elevation or peaks at intermediate elevations. An inverse relationship might be assumed for lichens and bryophytes, for which in forests richness has been shown to increase with elevation^27,28^. Our interest focuses on possible differences between taxonomic groups living in the same environment, as detected by Peters et al.^29^ in a big multi-taxon elevational study in the Afro-tropics who claimed for replication.

With regard to beta diversity, we hypothesize that, when increasing the elevational distance and looking at comparisons always starting from sample units at lowest elevation to all other elevations (along elevation diversity), turnover will increase due to a diversification of communities, as already found by Bishop et al.^30^ for ants. Accordingly, we assume the antagonistic component, along elevation nestedness, to decrease with elevation, since communities living on higher elevations usually are not subsets of low-land communities^20^.

We secondly hypothesize that neighboring elevational steps (e.g. comparison of sites between 2,000 and 2,500 m, i.e. step-wise diversity) show lower turnover when comparing sites of higher located steps, due to a more specific community formed by harsher abiotic environment^20,31^ and a generally lower species pool, producing more similar local communities^32,33^ Consequently, we assume step-wise nestedness to behave contrarily, being higher when comparing pairs of sites on higher located steps, due to a more similar, adapted community and the subsequent higher probability of species subsets.

To our knowledge, our work disentangles for the first time beta-diversity components turnover and nestedness on multiple levels along an elevational gradient for nine taxonomic groups. We aim at identifying common patterns for organisms sharing the same habitat, which is pastured grassland, and at best, our findings will be useful in delineating zones or belts mattering most for biodiversity conservation along an elevational gradient.

## Methods

### Study area

All species were sampled in 2016 within the LT(S)ER site Val Mazia/Matschertal (LTER_EU_IT_097, N 46.6840°, E 10.5860°, https://deims.org/11696de6-0ab9-4c94-a06b-7ce40f56c964), located in the Central Eastern Alps, in the northernmost part of Italy (Autonomous Province of Bolzano—South Tyrol). The continental climate results in approximately 525 mm mean annual precipitation and an average air temperature of 5.6 °C (1,925–2,005, at 1,580 m a.s.l., Hydrographic Office of the Province Bolzano, South Tyrol).

**Figure 1:**
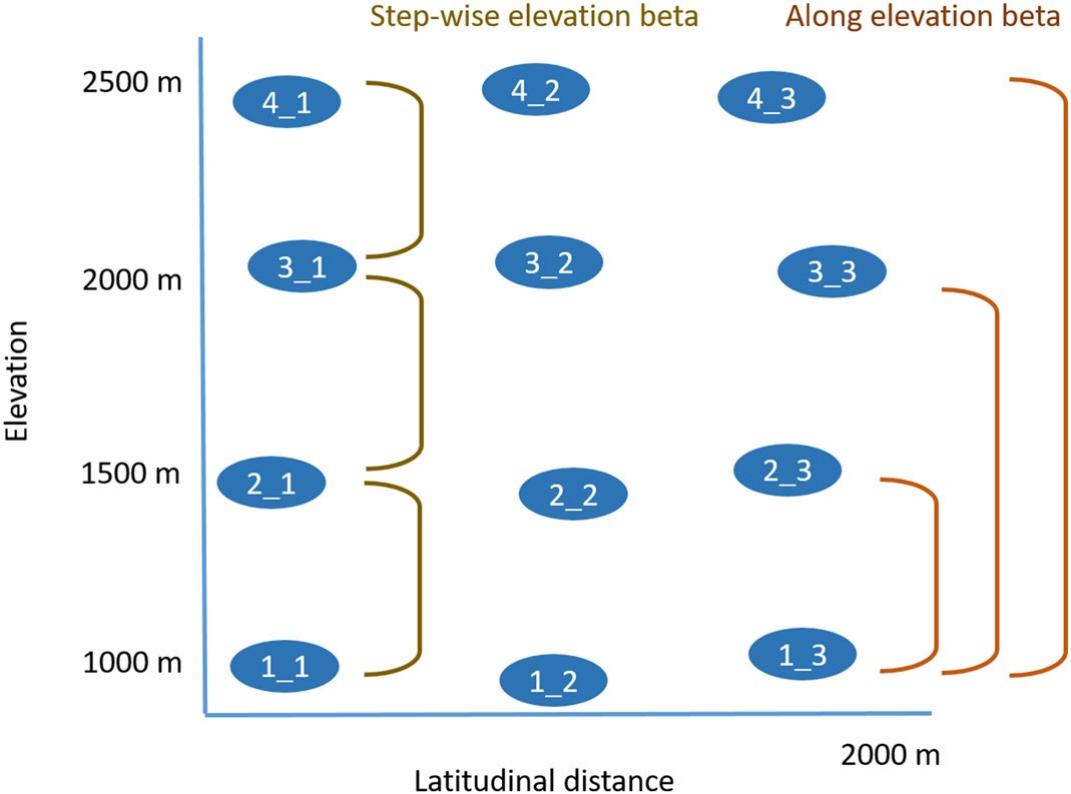
Study design with visualization of along and step-wise elevation beta diversity approach. Beta diversity components turnover and nestedness were calculated for all possible pairs and further distinguished between (i) along elevation beta diversity accounting for pairs starting from the lowest elevation to all other elevations (1,000–1,500, 1,000–2,000, 1,000–2,500 m a.s.l.), and (ii) step-wise beta diversity including pairs from one elevation to the next neighboring one (1,000–1,500, 1,500–2,000, 2,000–2,500 m). Each elevation was surveyed with three replicate sites (n total = 12).

Along a SW exposed, complete elevational gradient spanning from the lower montane zone (valley bottom) to the alpine zone (highest peak), we selected three replicate sites at four elevations each (1,000 m, 1,500, 2,000, 2,500, n total = 12, Fig. 1, site coordinates in Supplementary Table S1) within a horizontal distance of 2 km beeline.

All 12 sites have comparable slope (5–15°), are non-intensively grazed by cattle (0.5–1.5 livestock units per ha), are without additional fertilization or irrigation and were not subjected to substantial land-use-changes over the last 160 years^34^. The sites at the two lower elevation steps belong to an area which is protected by the Habitats Directive of the EU (code 6,240*)^35^.

### Field sampling

We recorded species presence and where possible abundance for nine taxonomic groups with standardized methods. For animal groups, we carefully met basic sampling conditions [cf.^36^] and always looked for exhaustiveness of species recording. A central point was established for each site wherefrom each group was sampled according to taxon-specific methods.

- Vascular plants were recorded from five random subplots (3 × 3 m) according to Elzinga et al.^37^ within an area of 531 m^2^ (radius from central point = 13 m). Species occurrence was recorded for each subplot.
- Bryophytes and lichens were recorded from five random subplots (50 × 40 cm) according to Elzinga et al.^37^ within an area of 531 m^2^. Species occurrence was recorded for each subplot. Specimens of species which were difficult to identify in the field were collected and identified in the laboratory.
- Grasshoppers including crickets and locusts were surveyed three times during summer period to ensure that all individuals were adult. They were caught by hand and with a sweep net for 20 min within 500 m^2^^38,39^, identified and released. Additionally, vocals were used to identify species.
- Butterflies and burnets were caught three times during summer period with a sweep net (45 min per site) within 500 m^2^^38,39^, identified and released.
- Earthworms were heat extracted from three soil core samples (20 × 20 cm, 15 cm deep, taken randomly within a 100 m^2^ area) with a modified Kempson apparatus^40^ according to Steinwandter et al.^33^. After extraction animals were identified to species level using a dissecting microscope.
- Beetles were taken from soil core samples (cf. earthworms) and caught with two pitfall traps (plastic cups with 8.5 cm diameter filled with 150 ml of propylene glycol) installed on opposing sides within a 5 m distance from the central point for two weeks [cf.^41^]. Individuals from pitfall traps and soil core samples were transferred to 75% ethanol and only adult specimens were identified to species level using a dissecting microscope.
- Spiders were collected by visual search, within soil core samples and pitfall traps (cf. beetles^42^). Individuals were transferred to 75% ethanol and only adult specimens were identified to species level using a dissecting microscope.
- Ants were recorded by counting nests within 4 m^2^ subplots and additionally collected within pitfall traps (cf. beetles) according to Schlick-Steiner et al.^43^. Individuals were collected and identified using a dissecting microscope.

### Data analyses

We first verified our sampling completeness by estimating sampling coverage (Table 1) with the function *iNEXT* within the R package inext^44^. Datatype was set to “incidence_freq” for bryophytes, lichen and vascular plants (due to data structure) and to “abundance” for all other taxa. In the case of incidence data, sample size refers to the number of sampling units, whereas for abundance data sample size equals the number of individuals in a sample^45^. Only a consistent and high sampling coverage estimation allows a compositional comparison of different taxonomic groups^46^.

**Table 1:** Results of mixed effect models testing the effect of elevational distance for overall beta (ß Sorensen) diversity and the components turnover and nestedness performed separately for along elevation and step-wise diversity for each taxonomic group.

Along elevation diversity							
Index	Taxon	AIC null model	AIC	Delta AIC	R^2^ m	m	P value
ß Sorensen	Lichens	− 78.822	− 77.181	− 1.641	0.009	0.304	0.549
	Bryophytes	− 34.596	− 43.130	8.534	0.212	0.566	0.001**
	Vascular plants	− 13.805	− 51.308	37.503	0.730	0.797	< 0.0001***
	Ants	10.912	− 8.088	18.999	0.531	0.531	< 0.0001***
	Spiders	− 17.266	− 34.348	17.082	0.463	0.564	< 0.0001***
	Grasshoppers	− 14.412	− 48.326	33.914	0.690	0.770	< 0.0001***
	Butterflies	− 16.009	− 29.811	13.802	0.404	0.513	< 0.0001***
	Beetles	− 42.217	− 55.775	13.558	0.373	0.535	< 0.0001***
Turnover	Lichens	− 36.598	− 36.790	0.192	0.049	0.423	0.139
	Bryophytes	6.428	2.467	3.961	0.177	0.296	0.015*
	Vascular plants	− 6.157	− 51.460	45.303	0.789	0.851	< 0.0001***
	Ants	11.461	− 9.031	20.492	0.556	0.556	< 0.0001***
	Spiders	4.405	− 9.809	14.214	0.341	0.597	< 0.0001***
	Grasshoppers	− 11.324	− 27.416	16.092	0.459	0.534	< 0.0001***
	Butterflies	5.122	− 12.575	17.697	0.476	0.580	< 0.0001***
	Beetles	− 31.561	− 48.266	16.705	0.482	0.522	< 0.0001***
Nestedness	Lichens	− 61.545	− 63.189	1.644	0.084	0.427	0.056
	Bryophytes	− 18.893	− 19.511	0.618	0.089	0.089	0.106
	Vascular plants	− 99.366	− 131.235	31.869	0.652	0.764	< 0.0001***
	Ants	− 144.290	− 147.810	3.520	0.179	0.179	0.0188*
	Spiders	− 44.919	− 48.912	3.993	0.117	0.532	0.0144*
	Grasshoppers	− 69.308	− 70.304	0.996	0.081	0.305	0.083
	Butterflies	− 40.931	− 52.181	11.250	0.378	0.385	0.0003***
	Beetles	− 96.174	− 108.200	12.030	0.321	0.543	0.0002***

Stepwise diversity							
Index	Taxon	AIC null model	AIC	Delta AIC	R^2^ m	R^2^ c	P value
ß Sorensen	Lichens	− 39.076	− 45.154	6.078	0.498	0.883	0.004**
	Bryophytes	− 6.970	− 10.837	3.867	0.255	0.396	0.015*
	Vascular plants	− 47.905	− 45.994	− 1.911	0.004	0.186	0.765
	Ants	3.638	− 1.357	4.995	0.344	0.559	0.008**
	Spiders	− 23.641	− 26.587	2.946	0.166	0.166	0.026*
	Grasshoppers	− 31.276	− 30.971	− 0.305	0.106	0.610	0.193
	Butterflies	− 31.647	− 32.699	1.052	0.136	0.330	0.081
	Beetles	− 49.135	− 48.248	− 0.887	0.044	0.193	0.292
Turnover	Lichens	− 15.138	− 22.681	7.543	0.587	0.959	0.002**
	Bryophytes	22.296	15.737	6.559	0.272	0.272	0.003**
	Vascular plants	− 45.773	− 45.991	0.218	0.080	0.155	0.136
	Ants	5.239	1.265	3.974	0.307	0.566	0.015*
	Spiders	3.753	5.305	− 1.552	0.016	0.059	0.503
	Grasshoppers	− 21.112	− 19.113	− 1.999	0.000	0.297	0.975
	Butterflies	− 10.993	− 9.788	− 1.204	0.050	0.569	0.372
	Beetles	− 21.388	− 19.401	− 1.987	0.000	0.000	0.909
Nestedness	Lichens	− 19.882	− 23.313	3.431	0.377	0.911	0.020*
	Bryophytes	0.697	0.702	− 0.005	0.090	0.312	0.158
	Vascular plants	− 115.490	− 122.950	7.460	0.461	0.652	0.002**
	Ants	− 144.290	− 147.890	3.600	0.182	0.182	0.018*
	Spiders	− 19.100	− 18.053	− 1.047	0.033	0.079	0.329
	Grasshoppers	− 64.643	− 67.487	2.844	0.296	0.716	0.028*
	Butterflies	− 35.734	− 33.734	− 2.000	0.000	0.774	0.990
	Beetles	− 41.188	− 40.529	− 0.659	0.047	0.047	0.247

AIC Null Model = is the AIC (Akaike information criterion) of the model without elevational distance as fixed factor, AIC = is the AIC of the model including elevational distance, Delta AIC is the difference of the applied model to the Null-Model. R^2^ m… marginal, R^2^ c… conditional. Significance levels: *...0.01–0.05, **...0.001–0.01, ***...0–0.001

Species richness was calculated by computing the cumulative number of species per site (n = 12) for each taxon separately. By applying the function *spline.plot* within the R package drsmooth^47^, we plotted a spline-estimated dose–response function on the actual richness data along the elevational gradient with its upper and lower 95 percent confidence bounds.

In order to have the same data base for all taxa, beta diversity was calculated using species occurrence data (not including abundance), which was then partitioned into turnover and nestedness components by applying the function *beta.pair* within the R package betapart^17^. This results in three matrices based on pair-wise comparisons of each site: the Sørensen dissimilarity index (β_sor_) expresses the total compositional variation with values ranging between 0 and 1, the Simpson dissimilarity index matrix (β_sim_) compositional changes due to species turnover, and β_sor_ minus β_sim_ is the resultant nestedness component β_sne_.

We further processed beta diversity data (β_sor,_ β_sim,_ β_sne_) to distinguish between (i) along elevation beta diversity (Fig. 1) accounting for pairs starting from the lowest elevation to all other elevations (1,000–1,500, 1,000–2,000, 1,000–2,500 m), and (ii) step-wise beta diversity (Fig. 1) including pairs from one elevation to the next neighboring one (1,000–1,500, 1,500–2,000, 2,000–2,500 m).

The effect of elevation on both step-wise and along beta diversity was tested with a linear mixed effects model using the R package lme4^48^. As fixed effects, we tested the elevational distance, as random effects we included the elevational replicate sites. We also tested for geographical autocorrelations in our dataset. To do so, we decomposed the total Euclidian distance between two sites into elevational distance (the variable to be tested) and the remaining distance (considered as potential spatial autocorrelation). We calculated this remaining distance by applying the Pythagorean theorem with elevation as one leg of a hypothetical triangle and the total distance as the hypothenuse^49^, and used it as fixed factor in the above explained model. Visual inspection of residual plots did not reveal any obvious deviations from homoscedasticity or normality. Significance of fixed factors was confirmed if Akaike Information Criterion^50^ was lowered by at least 2 points (AIC < 2) in comparison to the null model. Further, the R^2^ partitioned into marginal and conditional R^2^ was computed following Nakagawa and Schielzeth^51^. All analyses were conducted, and corresponding figures were produced using the open-source statistical programming language R (version 3.6.2, R Core Team^52^ in R Studio, version 1.1.383, RStudio Team^53^).

## Results

### Overall diversity and sampling coverage analyses

In total we detected 407 species comprising 42 lichens, 23 bryophytes, 166 vascular plants, 17 grasshoppers, 14 ants, 26 butterflies, 4 earthworms, 69 beetles, and 46 spiders (species list in Supplementary Table S2). Estimation of sampling coverage SC [cf.^46^] was very high for all elevations and taxa with a mean SC value of 0.95 (SD = 0.07). In few cases and only for singular elevations, sample-size-based R/E sampling curves, which compute diversity estimates for rarefied and extrapolated samples and plot the diversity estimates with respect to sample size^44,45^, did not flatten completely.

### Species richness

**Figure 2:**
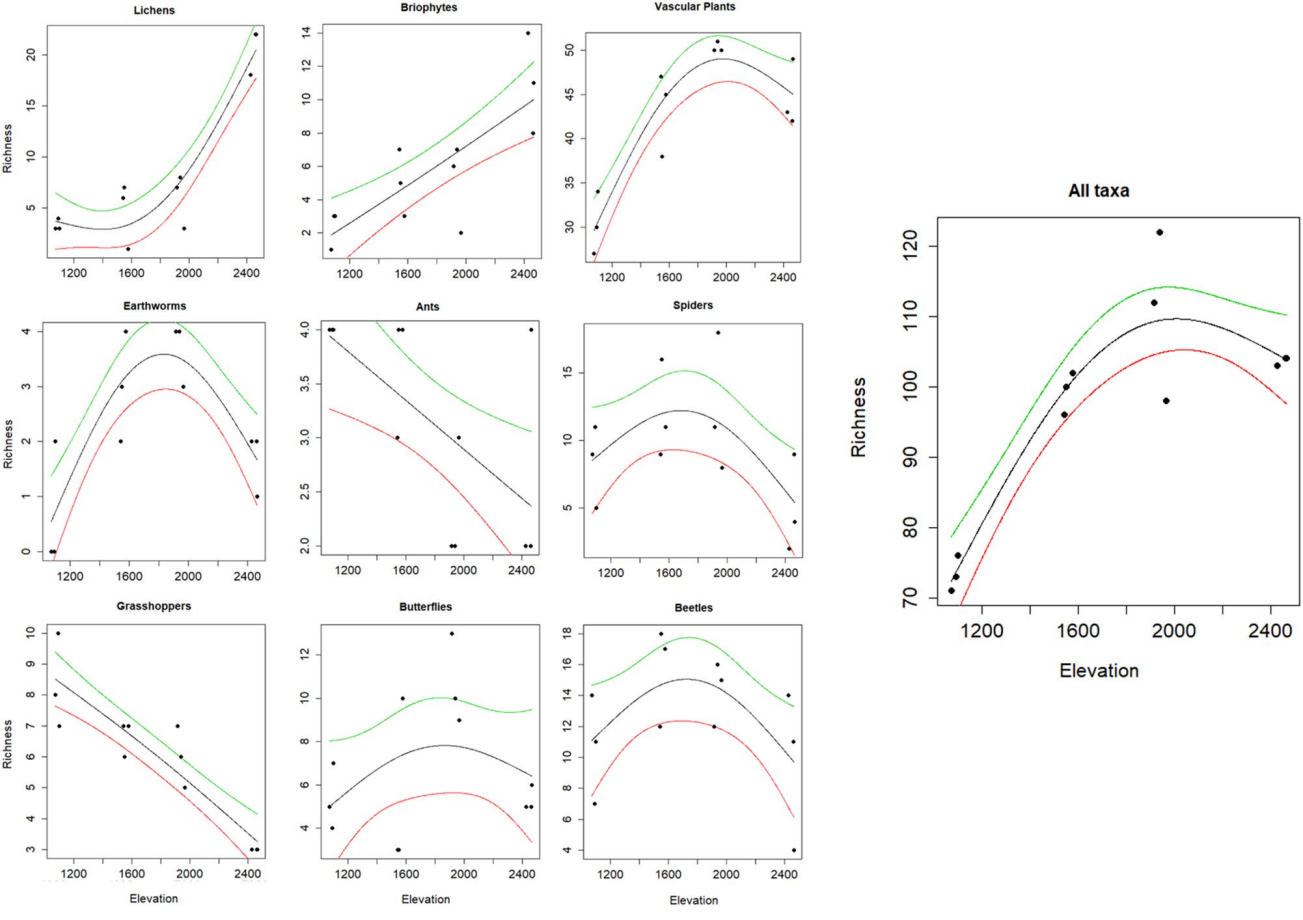
Relationship of species richness and elevation for 9 taxonomic groups. Cumulative number of species per site (n = 12) was calculated for each taxon separately and plotted with a spline-estimated dose–response function with upper (green line) and lower (red line) 95 percent confidence bounds. Figure was produced using R software (version 3.6.2, R Core Team, https://www.r-project.org/).

A hump-shaped relationship between elevation and species richness was detected for vascular plants, butterflies, beetles, spiders and earthworms, while species richness decreased linearly with elevation for grasshoppers and ants (Fig. 2). Species richness of lichens and bryophytes increased with increasing elevation. After this result, earthworms were excluded from all further analyses, since aside of very low species richness (gamma diversity equaled to only 4 species), on 1,000 and 2,500 m very few individuals were found.

### Beta diversity

Considering all possible pairs of comparisons of all sampled sites, the mean total beta diversity varied from 0.61 (grasshoppers) to 0.79 (lichens and ants). Partitioning total beta diversity and calculating portions, we found the highest percentage of turnover for vascular plants and ants (93.4 and 90.3, respectively), while nestedness was found to be highest for lichens (31.9%) and bryophytes (28.3%) for the investigated gradient.

**Figure 3:**
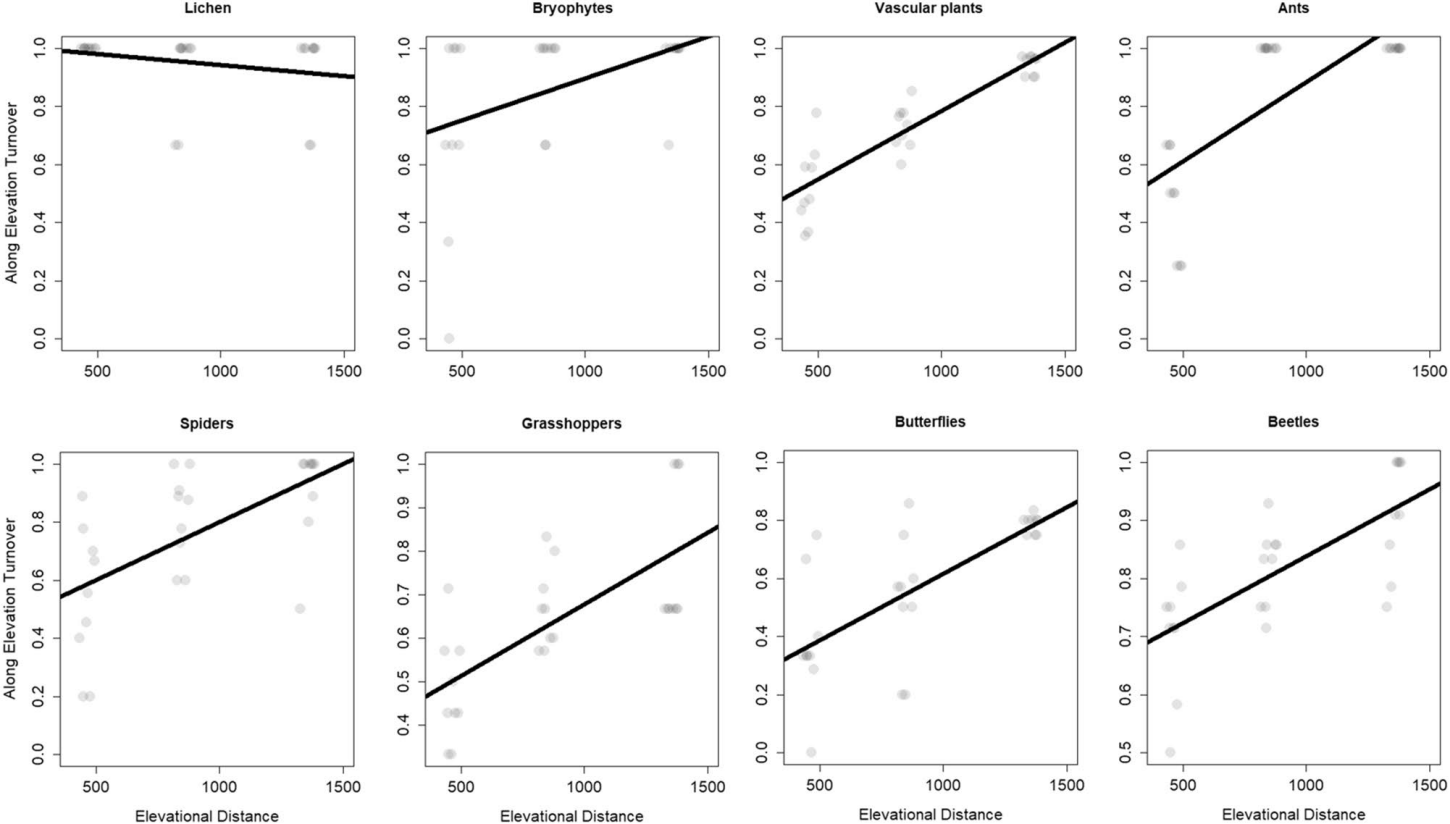
Along elevational turnover: the effect of elevational distance on turnover was tested with a linear mixed effects model. As fixed effects, we tested the elevational distance, as random effects the elevational replicate sites were modelled. The line indicates the response of turnover to elevational distance, grey points display turnover values of pair-wise comparisons. Figure was produced using R software (version 3.6.2, R Core Team, https://www.r-project.org/).

Along elevational turnover (all comparisons starting from the lowest elevation) increased significantly for all groups (increase of β_sim_ between 0.09 and 0.23), except for lichens (Table 1, Fig. 3).

**Figure 4:**
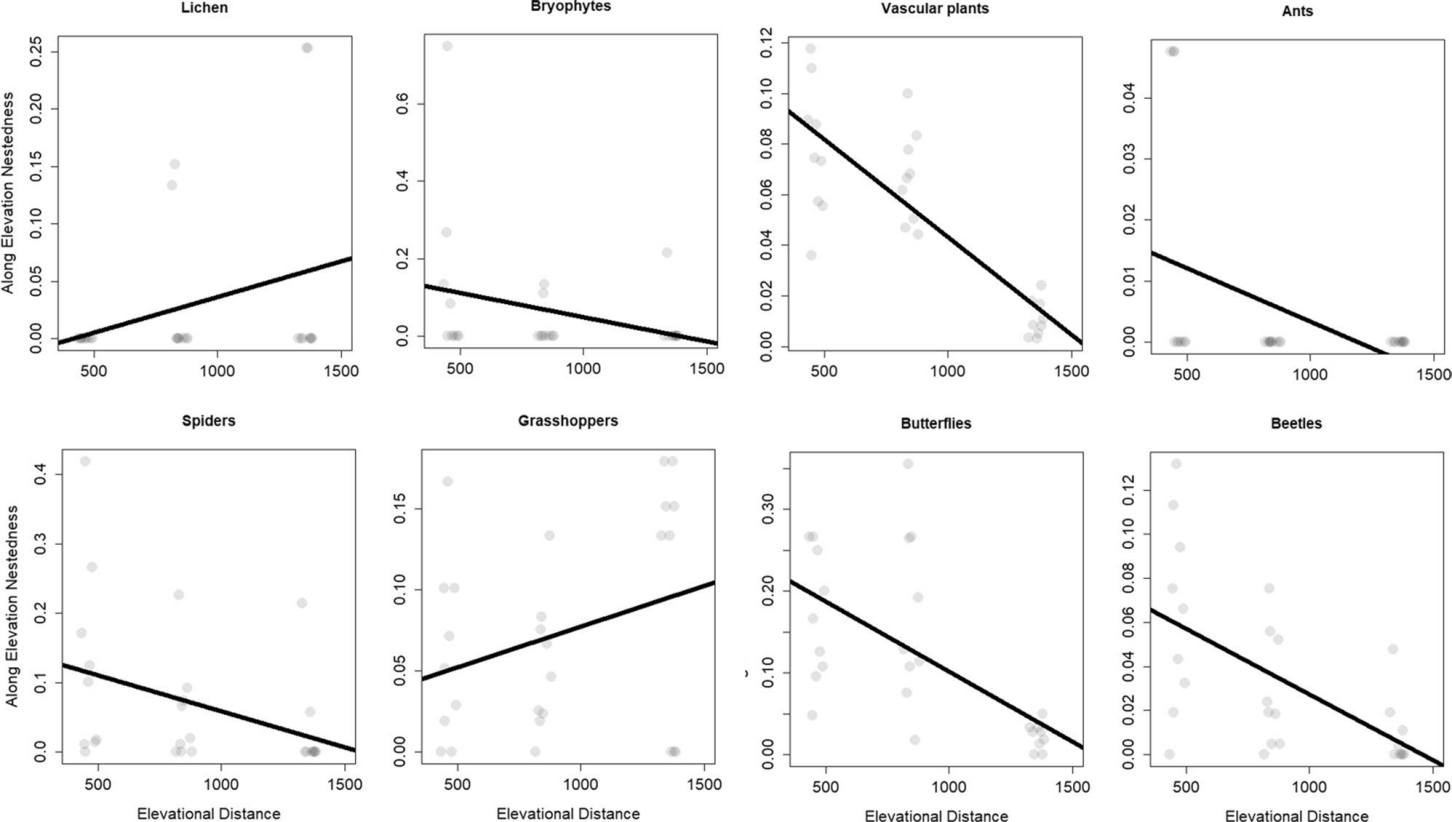
Along elevation nestedness: the effect of elevational distance on nestedness was tested with a linear mixed effects model. As fixed effects, we tested the elevational distance, as random effects the elevational replicate sites were modelled. The line indicates the response of nestedness to elevational distance, grey points display nestedness values of pair-wise comparisons. Figure was produced using R software (version 3.6.2, R Core Team, https://www.r-project.org/).

Nestedness decreased with increasing elevational distance for five groups (vascular plants, ants, butterflies, beetles, and spiders), while grasshoppers, bryophytes and lichens, showed no significant differences (Table 1, Fig. 4).

**Figure 5:**
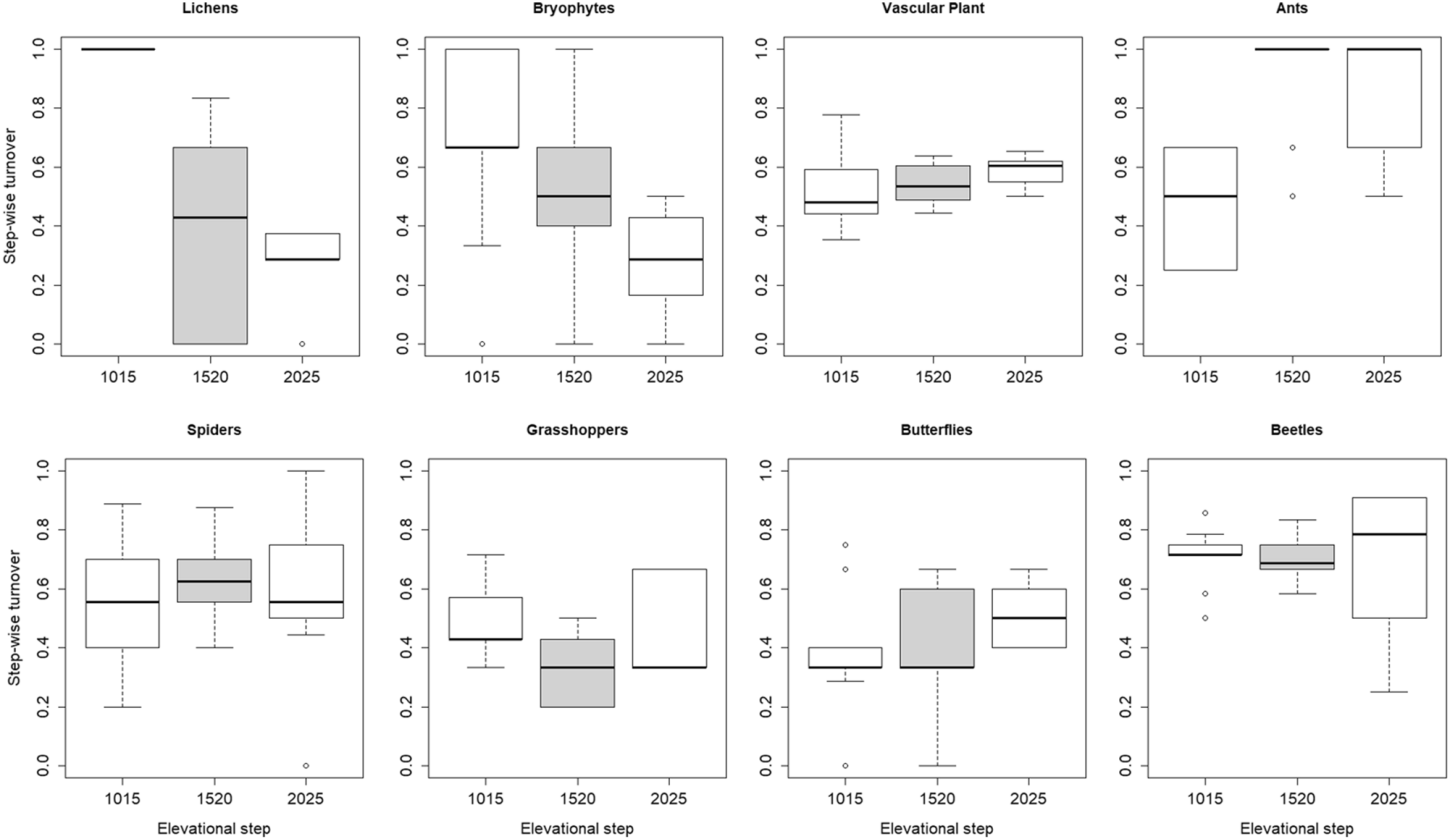
Step-wise turnover (pair-wise comparisons) of neighboring steps. 1,015… comparison of steps 1,000 and 1,500, 1,520… comparison of steps 1,500 and 2,000, 2,025… comparison of steps 2,000 and 2,500. Figure was produced using R software (version 3.6.2, R Core Team, https://www.r-project.org/).

**Figure 6:**
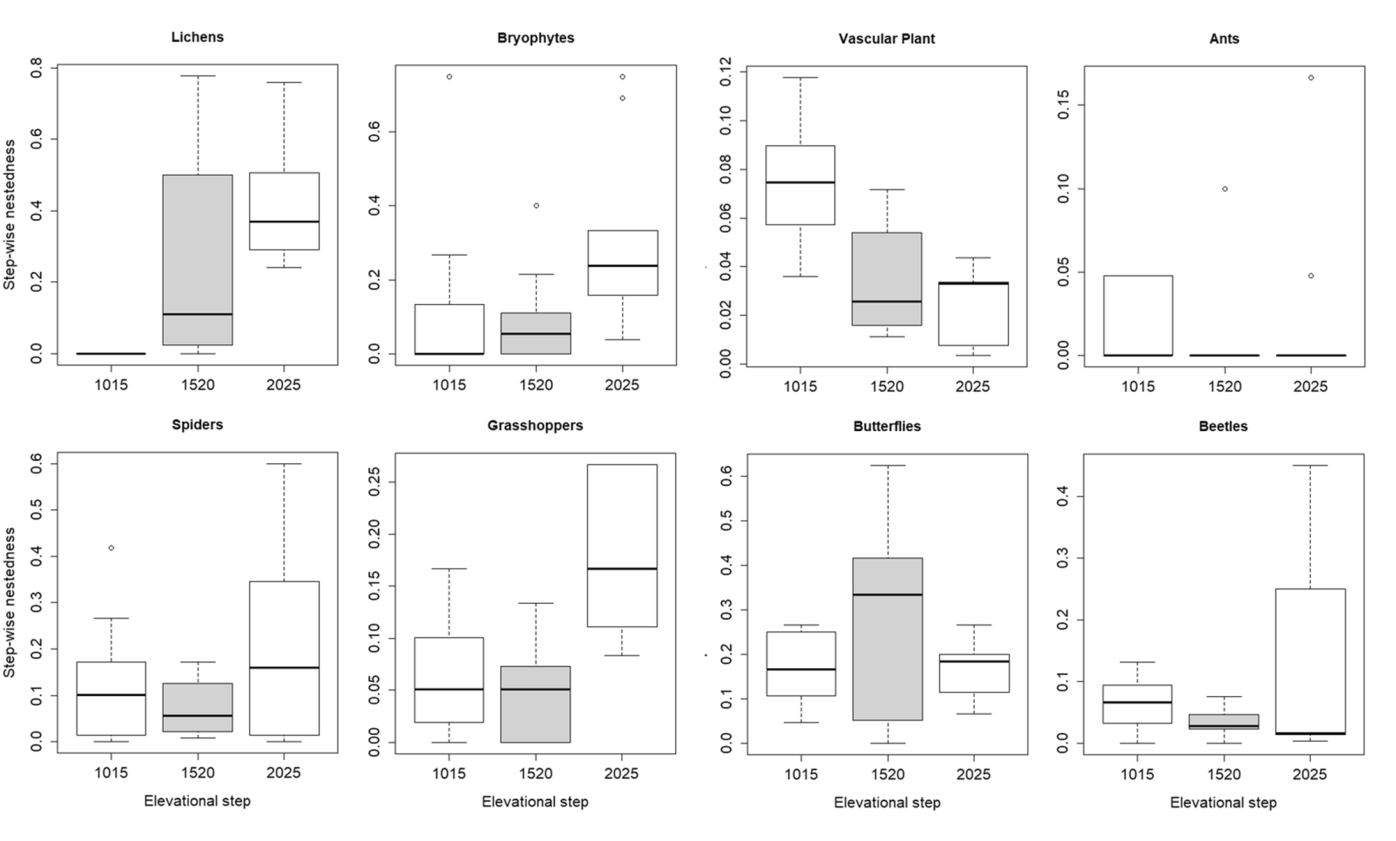
Step-wise nestedness (pair-wise comparisons) of neighboring steps. 1,015… comparison of steps 1,000 and 1,500, 1,520… comparison of steps 1,500 and 2,000, 2,025… comparison of steps 2,000 and 2,500. Figure was produced using R software (version 3.6.2, R Core Team, https://www.r-project.org/).

Regarding step-wise beta diversity only bryophytes and lichens had a significantly lower turnover and overall beta diversity for higher elevated steps, while all other groups showed no significant differences (Table 1, Fig. 5). Nestedness increased significantly with higher elevated steps with respect to grasshoppers and lichens, while results on vascular plants are opposed, featuring a significant lower nestedness at higher elevated steps (Table 1, Fig. 6). The other groups showed no significant differences. Out of the eight analyzed taxonomic groups, ants were the only ones showing a geographical autocorrelation for step-wise pair comparisons.

### Discussion

Our results provide a comprehensive overview of how diversity is organized in a vertical space for eight taxonomic groups (earthworms were excluded after first data analyses) comprising animals, plants, bryophytes, and lichens sharing the same habitat, namely pastured grasslands.

For the majority of the analyzed groups we detected common patterns, when looking at multi-taxonomic species richness and along elevational beta diversity. On the contrary, we found dissimilarities among taxonomic groups in the step-wise beta diversity.

With regard to species richness, it has been shown, that scale of spatial extent strongly impacts the shape of relationship between elevation and species richness: analyses of complete gradients most often lead to hump-shaped richness patterns, while omission of parts of the gradient at the upper and/or lower ends tends to favor monotonic increase or decrease with elevation^54^. According to that, with our study we captured the whole gradient for five out of our nine taxa, and the documented hump-shaped pattern is in line with previous work [e.g.^24,25,55^]. Apparently, the upper limit of the gradient may be missing for lichens and bryophytes and the lower limit for grasshoppers and ants (Fig. 2). Some studies on bryophytes performed in warmer climate zones found a hump-shaped relation of richness to elevation^56,57^. However, in the temperate zone, an increase of richness with elevation for lichens and bryophytes was found also by Nascimbene and Marini^27^ and Spitale^28^ who explained their results with a negative relationship of richness with temperature and associated conditions such as slower evaporation rates. Regarding grasshoppers and ants, the decrease of species richness with elevation was detected also by Peters et al.^29^, who attributed positive effects of available land areas on grasshoppers’ richness. Overall, Peters et al.^29^ argue that such unimodal species richness patterns hold only for single taxa analyses [cf.^58^] while when pooling a wider number of taxonomic groups, those patterns shift towards a decline of richness with elevation. We cannot confirm this finding derived from a study on Mt. Kilimanjaro, since our joint analysis of nine taxonomic groups still shows a hump-shape pattern (Fig. 2, in line with Viterbi et al.^24^). However, our elevational gradient is influenced by grazing and located in a completely different biogeographic region with other (temperate) climatic conditions, which may impact the shape of this relationship. We are also aware that particularly for animal groups, seasonality of sampling year or period may influence species recordings and hence total species pools of the study site might be larger. For our aim, which is not a species inventory of the sites, a snapshot embracing one season is still meaningful.

Beside species richness, which is one of the most frequently used indicators for biodiversity assessments for example when determining locally delimited spaces deserving protection, beta diversity is the key to scale up to the regional extent. The comparison of compositional differences between sites, further partitioned into turnover and nestedness according to Baselga^13^, allows the understanding of how biodiversity is assembled across elevations. As already found for some insect taxa [cf.^18^], turnover is the dominant component of beta diversity for all our studied taxa, which are vascular plants, lichen, mosses, ants, beetles, spiders, butterflies, and grasshoppers. According to our hypothesis that turnover will increase with increasing elevational distance, due to a diversification of communities, along elevational turnover (and total beta diversity i.e. ß Sorensen) increased significantly with increasing elevational distance for seven out of eight analyzed taxa. These results confirm the findings of studies focusing on single insect groups (e.g.^30^ for ants, ^20^ for dung-beetles, ^59^ for bees and wasps) and support the assumption that a considerable portion of species is restricted to certain zones and does not colonize entire gradients^30^. For along elevation nestedness, we assumed a decrease with elevation, since communities usually adapt to given conditions and probabilities for subsets between different elevations are lower. Five out of 8 analyzed support our hypothesis, consequently suggesting a differentiation of communities at some point(s), with low occurrences of subsets, as found for dung-beetles^20,55^. Our results underpin the assumption that mechanisms such as environmental filtering and dispersal limitations (reflected by high turnover rates) seem to prevail colonization and extinction patterns in community assembly of pastured grassland along temperate elevational gradients [cf.^59^].

When looking at similarities of neighboring steps (step-wise diversity), we could not confirm our second hypothesis assuming a decrease of total beta diversity and turnover when comparing sample units of higher located steps (due to abiotic constraints at mountain-tops promoting community speciation). The 8 analyzed groups do not show a congruent directional pattern – so far only two groups significantly decreased turnover (lichens and bryophytes) or increased nestedness (lichen and grasshopper) on higher elevated neighboring steps. The few other studies which analyzed beta diversity according to Baselga^13^ among elevational belts came to inconsistent findings. Paknia and Sh^31^ did not find a uniform pattern of beta diversity for moths along elevation, while da Silva et al.^20^ found a clustering of dung beetles into lowland (200–800 m) and highland (1,000–1,300 m) communities, driven by the turnover component. For vascular plants turnover was shown to be uneven along elevation and to peak between 1,800 and 2,200 m^60^. We assume that niche-breadth variation of some species^61^, barrier effects of treeline or ecotones^60^, or mobility of non-sessile groups like butterflies, beetles but also grasshoppers might influence turnover of species among neighboring elevational steps. Possibly, the range of our steps (500 m) is too large for detection of specifically adapted and hence similar communities at the upper limits of our gradient (2,000–2,500 m).

Summarizing along and step-wise diversity results, we conclude there is a significant change of species communities along elevation; however this transition seems not to be detectable between neighboring steps. This result could either be a hint for a rather slow transition of communities across the analyzed gradient, or for the existence of an elevational threshold that separates communities, which we were not able to identify with our approach (such as^59^).

### Conclusion

When unravelling spatial patterns of beta diversity, we found several studies focusing on arthropods in tropical to subtropical latitudes, while our study disentangles to our knowledge for the first time diversity of organisms belonging to different systematical classes in a temperate zone. The manifold possibilities to compare pairs of dissimilarities (e.g. within, along, step-wise, or complete gradient) and the frequent lack of specification of compared pairs present in literature, significantly hamper comparisons across studies and collective findings. However, our results confirm turnover to be the dominant component of beta diversity for all investigated taxa along the elevational gradient. Further, we detected a change of species communities for seven out of 8 analyzed taxa with increasing elevational distance but were not able to identify thresholds or delimitate zonal limits or boundaries of communities along the gradient. From a conservational point of view, this knowledge rather than delineating levels of major diversity losses, supports the idea that action sets or monitoring programs targeting at a capacious and preventive protection for species and their environment in mountainous regions require the consideration of entire gradients. Including information about specialist, rare or endemic species, niche breadth, or species traits into beta diversity assessments would significantly increase the future uncovering of spatial patterns of community assemblage.

## Supplementary information

Supplementary tables.

## Data Availability

All species recorded during this study are included in Supplementary Information files of this article.
