# Supplementary material for: Species richness and beta diversity patterns of multiple taxa along an elevational gradient in pastured grasslands in the European Alps
Source: Sci Rep. 2020 Jul 27;10:12516. doi: 10.1038/s41598-020-69569-9 (PMC7385172; doi:10.1038/s41598-020-69569-9)
Supplement: Supplementary file 1 — Supplementary tables. [file 41598_2020_69569_MOESM1_ESM.docx]

**Species richness and beta diversity patterns of multiple taxa along an elevational gradient in**

**pastured grasslands in the European Alps**

Veronika Fontana, Elia Guariento, Andreas Hilpold, Georg Niedrist, Michael Steinwandter, Daniel Spitale, Juri Nascimbene, Ulrike Tappeiner, Julia Seeber

Supplementary Table S1

**X and Y coordinates** of the 12 surveyed sites. Site_ID includes information on elevation.

| **site_ID** | **X Coordinate** | **Y Coordinate** |
| --- | --- | --- |
| 1000_1 | 10.57502 | 46.67743 |
| 1000_2 | 10.57619 | 46.67705 |
| 1000_3 | 10.57805 | 46.67546 |
| 1500_1 | 10.58048 | 46.68825 |
| 1500_2 | 10.58525 | 46.68433 |
| 1500_3 | 10.58563 | 46.68511 |
| 2000_1 | 10.57005 | 46.70306 |
| 2000_2 | 10.57002 | 46.70143 |
| 2000_3 | 10.59244 | 46.69169 |
| 2500_1 | 10.59386 | 46.70184 |
| 2500_2 | 10.59500 | 46.70280 |
| 2500_3 | 10.59796 | 46.70483 |

Supplementary Table S2

**Species List**

| Taxonomic group | Species |
| --- | --- |
| LICHENS | *Alectoria ochroleuca (Hoffm.) A. Massal.* |
| LICHENS | *Athallia pyracea (Ach.) Arup, Frödén & Søchting* |
| LICHENS | *Athallia saxifragarum (Poelt) Arup, Frödén & Søchting* |
| LICHENS | *Bryonora castanea (Hepp) Poelt* |
| LICHENS | *Bryoplaca sinapisperma (DC.) Søchting, Frödén & Arup* |
| LICHENS | *Caloplaca stillicidiorum (Vahl) Lynge var. muscorum (A. Massal.)* |
| LICHENS | *Candelariella commutata Otte & M. Westb.* |
| LICHENS | *Candelariella sp.* |
| LICHENS | *Cetraria aculeata (Schreb.) Fr.* |
| LICHENS | *Cetraria ericetorum Opiz* |
| LICHENS | *Cetraria islandica (L.) Ach. subsp. islandica* |
| LICHENS | *Cladonia arbuscula (Wallr.) Flot.* |
| LICHENS | *Cladonia coccifera gr.* |
| LICHENS | *Cladonia furcata (Huds.) Schrad. subsp. furcata* |
| LICHENS | *Cladonia furcata subsp. subrangiformis auct. non (Sandst.) Abbayes* |
| LICHENS | *Cladonia gracilis (L.) Willd.* |
| LICHENS | *Cladonia pyxidata (L.) Hoffm.* |
| LICHENS | *Cladonia rangiferina (L.) F.H. Wigg.* |
| LICHENS | *Cladonia rangiformis Hoffm.* |
| LICHENS | *Cladonia sp. (podezi a punta)* |
| LICHENS | *Cladonia sp. (squamule)* |
| LICHENS | *Cladonia uncialis (L.) F.H. Wigg. subsp. uncialis* |
| LICHENS | *Endocarpon pusillum Hedw.* |
| LICHENS | *Flavocetraria cucullata (Bellardi) Kärnefelt & A. Thell* |
| LICHENS | *Flavocetraria nivalis (L.) Kärnefelt & A. Thell* |
| LICHENS | *Fuscopannaria praetermissa (Nyl.) P.M. Jørg.* |
| LICHENS | *Lecidella wulfenii (Hepp) Körb.* |
| LICHENS | *Leptogium sp.* |
| LICHENS | *Megaspora verrucosa var. mutabilis (Ach.) Nimis & Cl. Roux* |
| LICHENS | *Parvoplaca tiroliensis (Zahlbr.) Arup, Søchting & Frödén* |
| LICHENS | *Peltigera didactyla (With.) J.R. Laundon* |
| LICHENS | *Peltigera lepidophora (Vain.) Bitter* |
| LICHENS | *Peltigera leucophlebia (Nyl.) Gyeln.* |
| LICHENS | *Peltigera rufescens (Weiss) Humb.* |
| LICHENS | *Peltigera sp.* |
| LICHENS | *Physconia muscigena (Ach.) Poelt var. muscigena* |
| LICHENS | *Placidium squamulosum (Ach.) Breuss* |
| LICHENS | *Psoroma tenue Henssen var. boreale Henssen* |
| LICHENS | *Rinodina sp.* |
| LICHENS | *Stereocaulon sp.* |
| LICHENS | *Thamnolia vermicularis (Sw.) Schaer. var. vermicularis* |
| LICHENS | *Xanthoparmelia stenophylla (Ach.) Ahti & D. Hawksw.* |
| BRYOPHYTES | *Abietinella abietina (Hedw.) M. Fleisch.* |
| BRYOPHYTES | *Brachythecium salebrosum (Hoffm. ex F. Weber & D. Mohr) Schimp.* |
| BRYOPHYTES | *Bryum argenteum Hedw.* |
| BRYOPHYTES | *Bryum caespiticium Hedw.* |
| BRYOPHYTES | *Campyliadelphus chrysophyllus (Brid.) Kanda* |
| BRYOPHYTES | *Ceratodon purpureus (Hedw.) Brid.* |
| BRYOPHYTES | *Desmatodon latifolius (Hedw.) Brid.* |
| BRYOPHYTES | *Dicranum spadiceum J.E. Zetterst.* |
| BRYOPHYTES | *Encalypta vulgaris Hedw.* |
| BRYOPHYTES | *Grimmia ovata F. Weber & D. Mohr* |
| BRYOPHYTES | *Hedwigia ciliata var leucophaea Bruch & Schimp.* |
| BRYOPHYTES | *Phascum cuspidatum Schreb. ex Hedw.* |
| BRYOPHYTES | *Polytrichum juniperinum Hedw.* |
| BRYOPHYTES | *Polytrichum piliferum Hedw.* |
| BRYOPHYTES | *Racomitrium canescens (Hedw.) Brid.* |
| BRYOPHYTES | *Rhytidium rugosum (Hedw.) Kindb.* |
| BRYOPHYTES | *Riccia sorocarpa Bisch.* |
| BRYOPHYTES | *Schistidium sp.* |
| BRYOPHYTES | *Sciuro-hypnum glaciale (Schimp.) Ignatov & Huttunen* |
| BRYOPHYTES | *Syntrichia ruralis (Hedw.) F. Weber & D. Mohr* |
| BRYOPHYTES | *Tortella tortuosa (Schrad. ex Hedw.) Limpr.* |
| BRYOPHYTES | *Weissia brachycarpa (Nees & Hornsch.) Jur.* |
| BRYOPHYTES | *Weissia longifolia Mitt.* |
| VASCULAR PLANTS | *Achillea millefolium agg. (L.)* |
| VASCULAR PLANTS | *Achillea moschata (Wulfen)* |
| VASCULAR PLANTS | *Achillea tomentosa (L.)* |
| VASCULAR PLANTS | *Aconitum napellus s.str. (L.)* |
| VASCULAR PLANTS | *Agrostis alpina (Scop.)* |
| VASCULAR PLANTS | *Agrostis capillaris (L.)* |
| VASCULAR PLANTS | *Alchemilla vulgaris agg. (L.)* |
| VASCULAR PLANTS | *Allium sphaerocephalon (L.)* |
| VASCULAR PLANTS | *Alyssum alyssoides (L.) L.* |
| VASCULAR PLANTS | *Antennaria dioica (L.) Gaertn.* |
| VASCULAR PLANTS | *Anthericum liliago (L.)* |
| VASCULAR PLANTS | *Anthoxanthum alpinum (Á. Löve & D. Löve)* |
| VASCULAR PLANTS | *Anthoxanthum odoratum (L.)* |
| VASCULAR PLANTS | *Anthyllis vulneraria (L.)* |
| VASCULAR PLANTS | *Arctostaphylos uva-ursi (L.) Spreng.* |
| VASCULAR PLANTS | *Arenaria serpyllifolia (L.)* |
| VASCULAR PLANTS | *Artemisia absinthium (L.)* |
| VASCULAR PLANTS | *Artemisia campestris (L.)* |
| VASCULAR PLANTS | *Aster alpinus (L.)* |
| VASCULAR PLANTS | *Astragalus exscapus (L.)* |
| VASCULAR PLANTS | *Astragalus onobrychis (L.)* |
| VASCULAR PLANTS | *Atocion rupestre (L.) Oxelman* |
| VASCULAR PLANTS | *Avenula praeusta (Rchb.)* |
| VASCULAR PLANTS | *Avenula pubescens (Huds.) Dumort.* |
| VASCULAR PLANTS | *Berberis vulgaris (L.)* |
| VASCULAR PLANTS | *Bothriochloa ischaemum (L.) Keng* |
| VASCULAR PLANTS | *Botrychium lunaria (L.) Sw.* |
| VASCULAR PLANTS | *Brachypodium rupestre (Host) Roem.&Schult.* |
| VASCULAR PLANTS | *Briza media (L.)* |
| VASCULAR PLANTS | *Bromus erectus (Huds.)* |
| VASCULAR PLANTS | *Bromus hordeaceus (L.)* |
| VASCULAR PLANTS | *Campanula rotundifolia (L.)* |
| VASCULAR PLANTS | *Campanula scheuchzeri (Vill.)* |
| VASCULAR PLANTS | *Carduus nutans (L.)* |
| VASCULAR PLANTS | *Carex caryophyllea (Latourr.)* |
| VASCULAR PLANTS | *Carex curvula (All.)* |
| VASCULAR PLANTS | *Carex ericetorum (Pollich)* |
| VASCULAR PLANTS | *Carex humilis (Leyss.)* |
| VASCULAR PLANTS | *Carex liparocarpos (Gaudin)* |
| VASCULAR PLANTS | *Carex sempervirens (Vill.)* |
| VASCULAR PLANTS | *Carex supina (Wahlenb.)* |
| VASCULAR PLANTS | *Carlina acaulis (L.)* |
| VASCULAR PLANTS | *Carlina vulgaris (L.)* |
| VASCULAR PLANTS | *Centaurea stoebe (L.)* |
| VASCULAR PLANTS | *Cerastium arvense (L.)* |
| VASCULAR PLANTS | *Cerastium holosteoides (Baumg.)* |
| VASCULAR PLANTS | *Cerastium semidecandrum (L.)* |
| VASCULAR PLANTS | *Chenopodium album (L.)* |
| VASCULAR PLANTS | *Chondrilla juncea (L.)* |
| VASCULAR PLANTS | *Cirsium acaule (Scop.)* |
| VASCULAR PLANTS | *Dianthus deltoides (L.)* |
| VASCULAR PLANTS | *Dianthus sylvestris (Wulfen)* |
| VASCULAR PLANTS | *Draba dubia (Suter)* |
| VASCULAR PLANTS | *Erigeron alpinus s.str. (L.)* |
| VASCULAR PLANTS | *Erigeron sp.* |
| VASCULAR PLANTS | *Erysimum rhaeticum (Hornem.) DC.* |
| VASCULAR PLANTS | *Euphorbia cyparissias (L.)* |
| VASCULAR PLANTS | *Euphrasia sp.* |
| VASCULAR PLANTS | *Festuca bauzanina (Pils) S. Arndt* |
| VASCULAR PLANTS | *Festuca guestfalica (s.lat.) (Boenn.)* |
| VASCULAR PLANTS | *Festuca halleri (All.)* |
| VASCULAR PLANTS | *Festuca nigrescens (Lam.)* |
| VASCULAR PLANTS | *Festuca pratensis s.str. (Huds.)* |
| VASCULAR PLANTS | *Festuca rubra agg. (L.)* |
| VASCULAR PLANTS | *Festuca rupicola (Heuff.)* |
| VASCULAR PLANTS | *Festuca valesiaca (Gaudin)* |
| VASCULAR PLANTS | *Fumana procumbens (Dunal) Gren. & Godr.* |
| VASCULAR PLANTS | *Galium anisophyllon (Vill.)* |
| VASCULAR PLANTS | *Galium pusillum agg. (L.)* |
| VASCULAR PLANTS | *Galium verum (L.)* |
| VASCULAR PLANTS | *Gentiana acaulis (L.)* |
| VASCULAR PLANTS | *Gentiana verna (L.)* |
| VASCULAR PLANTS | *Gentianella sp.* |
| VASCULAR PLANTS | *Helianthemum nummularium subsp. Obscurum (Čelak.) Holub* |
| VASCULAR PLANTS | *Helictotrichon versicolor (Vill.) Pilg.* |
| VASCULAR PLANTS | *Hieracium lactucella (Wallr.)* |
| VASCULAR PLANTS | *Hieracium pilosella s.lat. (inkl. H.velutinum) (L.)* |
| VASCULAR PLANTS | *Juncus trifidus (L.)* |
| VASCULAR PLANTS | *Juniperus communis subsp. Communis (L.)* |
| VASCULAR PLANTS | *Kobresia myosuroides (Vill.) Fiori* |
| VASCULAR PLANTS | *Koeleria hirsuta (Gaudin)* |
| VASCULAR PLANTS | *Koeleria pyramidata agg. (Lam.) P. Beauv.* |
| VASCULAR PLANTS | *Lappula squarrosa (Retz.) Dumort.* |
| VASCULAR PLANTS | *Larix decidua (Mill.)* |
| VASCULAR PLANTS | *Leontodon hispidus (L.)* |
| VASCULAR PLANTS | *Linum catharticum (L.)* |
| VASCULAR PLANTS | *Loiseleuria procumbens (L.) Desv.* |
| VASCULAR PLANTS | *Lotus corniculatus (L.)* |
| VASCULAR PLANTS | *Luzula campestris agg. (L.) DC.* |
| VASCULAR PLANTS | *Luzula lutea (All.)DC.* |
| VASCULAR PLANTS | *Luzula multiflora s.lat. (L.)* |
| VASCULAR PLANTS | *Luzula spicata (L.) DC.* |
| VASCULAR PLANTS | *Medicago falcata (L.) Arcang.* |
| VASCULAR PLANTS | *Minuartia gerardii (Willd.) Hayek* |
| VASCULAR PLANTS | *Minuartia laricifolia (L.) Schinz & Thell.* |
| VASCULAR PLANTS | *Minuartia recurva (All.) Schinz & Thell.* |
| VASCULAR PLANTS | *Minuartia sedoides (L.) Hiern Zwerg-Miere* |
| VASCULAR PLANTS | *Myosotis sylvatica agg. (Hoffm.)* |
| VASCULAR PLANTS | *Nardus stricta (L.)* |
| VASCULAR PLANTS | *Nigritella rhelicanii (Teppner & E. Klein)* |
| VASCULAR PLANTS | *Orobanche sp.* |
| VASCULAR PLANTS | *Petrorhagia saxifraga (L.) Link* |
| VASCULAR PLANTS | *Phleum phleoides (L.) H. Karst.* |
| VASCULAR PLANTS | *Phyteuma betonicifolium (Vill.)* |
| VASCULAR PLANTS | *Phyteuma hemisphaericum (L.)* |
| VASCULAR PLANTS | *Pinus sylvestris (L.)* |
| VASCULAR PLANTS | *Plantago lanceolata (L.)* |
| VASCULAR PLANTS | *Plantago strictissima (All.)* |
| VASCULAR PLANTS | *Poa alpina (L.)* |
| VASCULAR PLANTS | *Poa angustifolia (L.)* |
| VASCULAR PLANTS | *Poa molineri (Balb.)* |
| VASCULAR PLANTS | *Poa variegata (Lam.)* |
| VASCULAR PLANTS | *Polygala alpestris (Rchb.)* |
| VASCULAR PLANTS | *Polygala alpina (Poiret) Steud.* |
| VASCULAR PLANTS | *Polygala chamaebuxus (L.)* |
| VASCULAR PLANTS | *Polygonum viviparum (L.)* |
| VASCULAR PLANTS | *Potentilla aurea (L.)* |
| VASCULAR PLANTS | *Potentilla crantzii (Crantz) Fritsch* |
| VASCULAR PLANTS | *Potentilla pusilla (Host)* |
| VASCULAR PLANTS | *Prunella vulgaris (L.)* |
| VASCULAR PLANTS | *Pseudoturritis turrita (L.) Al-Shehbaz* |
| VASCULAR PLANTS | *Pulsatilla montana (Hoppe) Rchb.* |
| VASCULAR PLANTS | *Pulsatilla vernalis (L.) Mill.* |
| VASCULAR PLANTS | *Ranunculus bulbosus (L.)* |
| VASCULAR PLANTS | *Ranunculus montanus agg. (Willd.)* |
| VASCULAR PLANTS | *Saponaria ocymoides (L.)* |
| VASCULAR PLANTS | *Saxifraga exarata (Vill.)* |
| VASCULAR PLANTS | *Saxifraga paniculata (Mill.)* |
| VASCULAR PLANTS | *Scabiosa triandra (L.)* |
| VASCULAR PLANTS | *Scleranthus annuus agg. (L.)* |
| VASCULAR PLANTS | *Scorzoneroides helvetica (Mérat) Holub* |
| VASCULAR PLANTS | *Securigera varia (L.) Lassen* |
| VASCULAR PLANTS | *Sedum rupestre agg. (L.) P.V.Heath* |
| VASCULAR PLANTS | *Sedum sexangulare (L.)* |
| VASCULAR PLANTS | *Sempervivum arachnoideum (L.)* |
| VASCULAR PLANTS | *Sempervivum montanum (L.)* |
| VASCULAR PLANTS | *Sempervivum tectorum (Griseb. & Schenk) L.* |
| VASCULAR PLANTS | *Senecio abrotanifolius subsp. Tiroliensis (A.Kern.) Gams* |
| VASCULAR PLANTS | *Senecio carniolicus s.lat. (Willd.) Schrank* |
| VASCULAR PLANTS | *Silene acaulis s.lat. (L.) Jacq.* |
| VASCULAR PLANTS | *Silene nutans (L.)* |
| VASCULAR PLANTS | *Silene otites (L.) Wibel* |
| VASCULAR PLANTS | *Stipa capillata (L.)* |
| VASCULAR PLANTS | *Stipa pennata (L.)* |
| VASCULAR PLANTS | *Taraxacum cf. alpestre agg. (Tausch)* |
| VASCULAR PLANTS | *Taraxacum officinale agg. (L.) Weber ex F.H. Wigg* |
| VASCULAR PLANTS | *Teucrium chamaedrys (L.)* |
| VASCULAR PLANTS | *Teucrium montanum (L.)* |
| VASCULAR PLANTS | *Thesium alpinum (L.)* |
| VASCULAR PLANTS | *Thesium linophyllon (L.)* |
| VASCULAR PLANTS | *Thymus praecox (Opiz)* |
| VASCULAR PLANTS | *Thymus sp.* |
| VASCULAR PLANTS | *Trifolium alpestre (L.)* |
| VASCULAR PLANTS | *Trifolium alpinum (L.)* |
| VASCULAR PLANTS | *Trifolium arvense (L.)* |
| VASCULAR PLANTS | *Trifolium campestre (Schreb.)* |
| VASCULAR PLANTS | *Trifolium montanum (L.)* |
| VASCULAR PLANTS | *Trifolium pratense (L.)* |
| VASCULAR PLANTS | *Ulmus pumila (L.)* |
| VASCULAR PLANTS | *Verbascum lychnitis (L.)* |
| VASCULAR PLANTS | *Veronica chamaedrys (L.)* |
| VASCULAR PLANTS | *Veronica fruticans (Jacq.)* |
| VASCULAR PLANTS | *Veronica spicata (L.)* |
| VASCULAR PLANTS | *Viola canina (L.)* |
| VASCULAR PLANTS | *Viola rupestris (F.W. Schmidt)* |
| VASCULAR PLANTS | *Viola sp.* |
| GRASSHOPPERS | *Calliptamus italicus (Linnaeus)* |
| GRASSHOPPERS | *Chorthippus biguttulus (Linnaeus)* |
| GRASSHOPPERS | *Chorthippus mollis (Charpentier)* |
| GRASSHOPPERS | *Chorthippus parallelus (Zetterstedt)* |
| GRASSHOPPERS | *Gomphocerus sibiricus (Linnaeus)* |
| GRASSHOPPERS | *Oedipoda caerulescens (Linnaeus)* |
| GRASSHOPPERS | *Omocestus haemorrhoidalis (Charpentier)* |
| GRASSHOPPERS | *Omocestus petraeus (Brisout de Barneville)* |
| GRASSHOPPERS | *Omocestus viridulus (Linnaeus)* |
| GRASSHOPPERS | *Psophus stridulus (Linnaeus)* |
| GRASSHOPPERS | *Stenobothrus lineatus (Panzer)* |
| GRASSHOPPERS | *Stenobothrus nigromaculatus (Herrich-Schäffer)* |
| GRASSHOPPERS | *Oecanthus pellucens (Scopoli)* |
| GRASSHOPPERS | *Tetrix bipunctata bipunctata (Linnaeus)* |
| GRASSHOPPERS | *Decticus verrucivorus (Linnaeus, 1758)* |
| GRASSHOPPERS | *Platycleis albopunctata grisea (Fabricius)* |
| GRASSHOPPERS | *Roeseliana roeselii (Hagenbach)* |
| ANTS | *Formica clara (Forel)* |
| ANTS | *Formica lemani (Bondroit)* |
| ANTS | *Formica rufibarbis (Fabricius)* |
| ANTS | *Formica sanguinea (Latreille)* |
| ANTS | *Lasius meridionalis (Bondroit)* |
| ANTS | *Lasius psammophilus (Seifert)* |
| ANTS | *Leptothorax acervorum (Fabricius)* |
| ANTS | *Solenopsis fugax (Latreille)* |
| ANTS | *Temnothorax interruptus (Schenck)* |
| ANTS | *Temnothorax nigirceps (Mayr)* |
| ANTS | *Temnothorax tuberum (Fabricius)* |
| ANTS | *Tetramorium alpestre (Steiner, Schlick-Steiner & Seifert)* |
| ANTS | *Tetramorium impurum (Foerster)* |
| ANTS | *Tetramorium indocile (Santschi)* |
| BUTTERFLIES | *Pyrgus carthami (Hubner)* |
| BUTTERFLIES | *Pyrgus serratulae (Rambur)* |
| BUTTERFLIES | *Aricia artaxerxes (Fabricius)* |
| BUTTERFLIES | *Cyaniris semiargus (Rottemburg)* |
| BUTTERFLIES | *Lysandra bellargus (Rottemburg)* |
| BUTTERFLIES | *Plebejus argus (Linnaeus)* |
| BUTTERFLIES | *Plebejus optilete (Knoch)* |
| BUTTERFLIES | *Plebejus trappi (Verity)* |
| BUTTERFLIES | *Polyommatus icarus (Rottemburg)* |
| BUTTERFLIES | *Aglais urticae (Linnaeus)* |
| BUTTERFLIES | *Argynnis aglaja (Linnaeus)* |
| BUTTERFLIES | *Chazara briseis (Linnaeus)* |
| BUTTERFLIES | *Coenonympha pamphilus (Linnaeus)* |
| BUTTERFLIES | *Erebia alberganus (de Prunner)* |
| BUTTERFLIES | *Erebia medusa (Denis & Schiffermuller)* |
| BUTTERFLIES | *Erebia tyndarus (Esper)* |
| BUTTERFLIES | *Hipparchia semele (Linnaeus)* |
| BUTTERFLIES | *Maniola jurtina (Linnaeus)* |
| BUTTERFLIES | *Melanargia galathea (Linnaeus)* |
| BUTTERFLIES | *Melitaea cinxia (Linnaeus)* |
| BUTTERFLIES | *Melitaea varia (Meyer-Dur)* |
| BUTTERFLIES | *Melitea athalia (Rottemburg)* |
| BUTTERFLIES | *Vanessa cardui (Linnaeus)* |
| BUTTERFLIES | *Gonepteryx rhamni (Linnaeus)* |
| BUTTERFLIES | *Zygaena carniolica (Scopoli)* |
| BUTTERFLIES | *Zygaena purpuralis (Brunnich)* |
| EARTHWORMS | *Aporrectodea rosea (Savigny)* |
| EARTHWORMS | *Dendrobaena octaedra (Savigny)* |
| EARTHWORMS | *Lumbricus rubellus (Hoffmeister)* |
| EARTHWORMS | *Octolasion lacteum (Orley)* |
| BEETLES | *Catapion seniculus (Kirby)* |
| BEETLES | *Squamapion atomarium (Kirby)* |
| BEETLES | *Amara aenea (DeGeer)* |
| BEETLES | *Amara infima (Duftschmid)* |
| BEETLES | *Amara lunicollis Schiødte)* |
| BEETLES | *Badister bullatus (Schrank)* |
| BEETLES | *Calathus fuscipes (Goeze)* |
| BEETLES | *Carabus convexus (Fabricius)* |
| BEETLES | *Dyschirius globosus (Herbst)* |
| BEETLES | *Harpalus anxius (Duftschmid)* |
| BEETLES | *Harpalus pumilus (Sturm)* |
| BEETLES | *Harpalus rubripes (Duftschmid)* |
| BEETLES | *Harpalus smaragdinus (Duftschmid)* |
| BEETLES | *Pterostichus jurinei (Panzer)* |
| BEETLES | *Pterostichus unctulatus (Duftschmid)* |
| BEETLES | *Syntomus truncatellus (Linnaeus)* |
| BEETLES | *Cassida margaritacea (Schaller)* |
| BEETLES | *Chaetocnema hortensis (Geoffroy)* |
| BEETLES | *Longitarsus luridus (Scopoli)* |
| BEETLES | *Longitarsus pratensis (Panzer)* |
| BEETLES | *Opetiopalpus sabulosus (Motschulsky)* |
| BEETLES | *Coccinella septempunctata (Linnaeus)* |
| BEETLES | *Hippodamia variegata (Goeze)* |
| BEETLES | *Atomaria analis (Erichson)* |
| BEETLES | *Cathormiocerus aristatus (Gyllenhal)* |
| BEETLES | *Rhinoncus pericarpius (Linnaeus)* |
| BEETLES | *Romualdius scaber (Linnaeus)* |
| BEETLES | *Sibinia tibialis (Gyllenhal)* |
| BEETLES | *Sitona sulcifrons argutulus (Gyllenhal)* |
| BEETLES | *Strophosoma faber (Herbst)* |
| BEETLES | *Trachyphloeus heymesi (Hubentha)* |
| BEETLES | *Trachyphloeus rectus (C.G.Thomson)* |
| BEETLES | *Trachyphloeus spinimanus (Germar)* |
| BEETLES | *Tychius aureolus (Kiesenwetter)* |
| BEETLES | *Tychius picirostris (Germar)* |
| BEETLES | *Tychius squamulatus (Gyllenhal)* |
| BEETLES | *Agrypnus murinus (Linnaeus)* |
| BEETLES | *Clanoptilus affinis (Ménétriés)* |
| BEETLES | *Aphodius abdominalis (Bonelli)* |
| BEETLES | *Aphodius haemorroidalis (Linnaeus)* |
| BEETLES | *Aphodius rufipes (Linnaeus)* |
| BEETLES | *Diastictus vulneratus (Sturm)* |
| BEETLES | *Onthophagus fracticornis (Preyssler)* |
| BEETLES | *Protaetia cuprea (Fabricius)* |
| BEETLES | *Phylloperta horticola (Linnaeus)* |
| BEETLES | *Aleochara maculata (Brisout de Barneville)* |
| BEETLES | *Amischa analis (Gravenhorst)* |
| BEETLES | *Anaulacaspis nigra (Gravenhorst)* |
| BEETLES | *Astenus gracialis (Paykull)* |
| BEETLES | *Atheta tibialis (Heer9)* |
| BEETLES | *Liogluta longiuscula (Gravenhorst)* |
| BEETLES | *Ocypus picipennis fallaciosus (G.Müller)* |
| BEETLES | *Oligota inexspectata (Williams & S.A.)* |
| BEETLES | *Ontholestes haroldi (Eppelsheim)* |
| BEETLES | *Oxypoda togata (Erichson)* |
| BEETLES | *Philonthus carbonarius (Gravenhorst)* |
| BEETLES | *Philonthus cognatus (Stephens)* |
| BEETLES | *Philonthus decorus (Gravenhorst)* |
| BEETLES | *Philonthus frigidus (Märkel & Kiesenwetter)* |
| BEETLES | *Philonthus lepidus (Gravenhorst)* |
| BEETLES | *Philonthus nimbicola (Fauvel)* |
| BEETLES | *Quedius paradisianus (Heer)* |
| BEETLES | *Tachinus corticinus (Gravenhorst)* |
| BEETLES | *Tachinus rufipes (Linnaeus)* |
| BEETLES | *Tachyporus dispar (Paykull)* |
| BEETLES | *Tachyporus scitulus (Erichson)* |
| BEETLES | *Xantholinus linearis (Olivier & A.G.)* |
| BEETLES | *Opatrum sabulosum (Linnaeus)* |
| BEETLES | *Orthocerus clavicornis (Linnaeus)* |
| SPIDERS | *Hypososinga albovittata (Westring)* |
| SPIDERS | *Cheiracanthium punctorium (Villers)* |
| SPIDERS | *Altella subnigra (O. Pickard-Cambridge)* |
| SPIDERS | *Drassodes lapidosus (Walckenaer)* |
| SPIDERS | *Drassodes pubescens (Thorell)* |
| SPIDERS | *Drassyllus praeficus (L. Koch)* |
| SPIDERS | *Drassyllus pusillus (C. L. Koch)* |
| SPIDERS | *Gnaphosa muscorum (L. Koch)* |
| SPIDERS | *Haplodrassus dalmatensis (L. Koch)* |
| SPIDERS | *Haplodrassus signifer (C. L. Koch)* |
| SPIDERS | *Micaria fulgens (Walckenaer)* |
| SPIDERS | *Parasyrisca vinosa (Simon)* |
| SPIDERS | *Zelotes electus (C. L. Koch)* |
| SPIDERS | *Caracladus avicula (L. Koch)* |
| SPIDERS | *Dicymbium nigrum brevisetosum (Locket)* |
| SPIDERS | *Erigone dentipalpis (Wider)* |
| SPIDERS | *Maso sundevalli (Westring)* |
| SPIDERS | *Mermessus trilobatus (Emerton)* |
| SPIDERS | *Pelecopsis parallela (Wider)* |
| SPIDERS | *Tapinocyba pallens (O. Pickard-Cambridge)* |
| SPIDERS | *Tiso vagans (Blackwall)* |
| SPIDERS | *Trichoncus affinis (Kulczyński)* |
| SPIDERS | *Trichopertna cito (O. Pickard-Cambridge)* |
| SPIDERS | *Alopecosa cuneata (Clerck)* |
| SPIDERS | *Alopecosa trabalis (Clerck)* |
| SPIDERS | *Pardosa bifasciata (C. L. Koch)* |
| SPIDERS | *Pardosa blanda (C. L. Koch)* |
| SPIDERS | *Pardosa monticola (Clerck)* |
| SPIDERS | *Pardosa palustris (Linnaeus)* |
| SPIDERS | *Thanatus arenarius (L. Koch)* |
| SPIDERS | *Thanatus coloradensis (Keyserling)* |
| SPIDERS | *Thanatus oblongiusculus (Lucas)* |
| SPIDERS | *Aelurillus v-insignitus (Clerck)* |
| SPIDERS | *Asianellus festivus (C. L. Koch)* |
| SPIDERS | *Pellenes tripunctatus (Walckenaer)* |
| SPIDERS | *Phlegra fasciata (Hahn)* |
| SPIDERS | *Talavera petrensis (C. L. Koch)* |
| SPIDERS | *Asagena phalerata (Panzer)* |
| SPIDERS | *Enoplognatha thoracica (Hahn)* |
| SPIDERS | *Steatoda albomaculata (De Geer)* |
| SPIDERS | *Xysticus audax (Schrank)* |
| SPIDERS | *Xysticus cristatus (Clerck)* |
| SPIDERS | *Xysticus erraticus (Blackwall)* |
| SPIDERS | *Xysticus ninnii (Thorell)* |
| SPIDERS | *Zodarion rubidum (Simon)* |
| SPIDERS (HARVESTMEN) | *Mitopus morio (Fabricius)* |
|  |  |
|  |  |
